# Gold Nanoparticles-Induced Modifications in Cell Wall Composition in Barley Roots

**DOI:** 10.3390/cells10081965

**Published:** 2021-08-02

**Authors:** Anna Milewska-Hendel, Katarzyna Sala, Weronika Gepfert, Ewa Kurczyńska

**Affiliations:** Faculty of Natural Sciences, Institute of Biology, Biotechnology and Environmental Protection, University of Silesia in Katowice, Jagiellońska 28, 40-032 Katowice, Poland; katarzyna.sala@us.edu.pl (K.S.); witekika@gmail.com (W.G.)

**Keywords:** abiotic stress, AGPs, barley, gold nanoparticles, immunohistochemistry, pectin, root development

## Abstract

The increased use of nanoparticles (NP) in different industries inevitably results in their release into the environment. In such conditions, plants come into direct contact with NP. Knowledge about the uptake of NP by plants and their effect on different developmental processes is still insufficient. Our studies concerned analyses of the changes in the chemical components of the cell walls of *Hordeum vulgare* L. roots that were grown in the presence of gold nanoparticles (AuNP). The analyses were performed using the immunohistological method and fluorescence microscopy. The obtained results indicate that AuNP with different surface charges affects the presence and distribution of selected pectic and arabinogalactan protein (AGP) epitopes in the walls of root cells.

## 1. Introduction

Plants live in diverse environments and nature, they are exposed to multiple abiotic stress factors, some of which are predicted to increase in severity with the advances in science and technology. The recent rapid progress of nanotechnology, in addition to its benefits, might also be an invisible danger to living organisms, including plants. Nanomaterials (NM) are frequently used in agriculture, the industrial sector, biomedicine, cosmetics, drug delivery, or material science [1,2,3]. This huge production of NM has led to their release into the environment, which affects the entire ecosystem [4]. When they enter crops, NM also poses a threat to the human population by contaminating the food chain. This has drawn the attention of many research groups who investigate the potential effects of nanoparticles (NP) on plants [5,6,7,8,9]. At present, nanotoxicology is a new, widely studied field of research. To date, it has been well documented that plants can uptake and accumulate NP, which could cause histological, morphological, physiological, and genotoxic changes in the plant system [10,11,12,13,14,15,16,17,18].

The effect of NP on plants depends on their composition, concentration, size, shape, and surface charge as well as the plant species [16,19,20]. One of the most important properties that affects the uptake of NP and their effect on plants particle is size. In theory, NP and their aggregates can be taken up by plant tissues if they are smaller than the pores of the cell wall, which are usually 3.3–6.2 nm [21,22,23,24]. Research on copper oxide (CuO) NP of various sizes (25, 50, and 250 nm) on *Glycine max* revealed that 25 nm NP can cross cell wall barriers and cause higher oxidative stress. In comparison, 250 nm NP has less surface reactivity and, therefore, lower toxicity [25]. Another survey found that natural organic matter that is coated with carbon nanotubes 240 nm in diameter exhibited no uptake. In comparison, fullerene C70, which has a diameter of 1.2 nm, penetrated roots and subsequently moved to the stem, leaves, and seeds of a rice plant [26]. An experiment on tobacco revealed that 3.5 nm gold nanoparticles (AuNP) penetrated the roots, while 18 nm AuNP remained agglomerated on the root surface [27]. Studies on barley demonstrated that AuNP of various sizes (5 and 20 nm) did not enter the roots but rather accumulated on their surface [22]. Another critical factor that affects the uptake and potential toxicity of plants is the surface charge of the NP [28,29]. A comprehensive investigation on different species found that positively-charged AuNP were more absorbed on the root surface and that their content in the roots was higher than negatively-charged AuNP. Therefore, the internalization rate and transport efficiency of positively-charged NP were much lower than those of negatively-charged NP [30]. Another study also showed that positively-charged cerium oxide (CeO_2_) NP were more prone to absorb on the root surface, while negatively-charged NP had a greater ability to migrate inside tomatoes [31]. Research on Arabidopsis revealed that regardless of the surface charge, AuNP did not enter the roots but were accumulated on the root surface. Moreover, AuNP affected the root histology and ultrastructure differently, which was dependent on AuNP surface properties [1]. Neutral 5 nm AuNP did not affect the barley root morphology [2], but the application of positively charged 5 nm AuNP resulted in the development of a “hairless” phenotype of barley roots [3]. These findings indicate that the physicochemical properties of NP are of great importance for their effect on plants.

One of the crucial aspects of the research on plant-NP interactions is the study of the cell wall that comes into contact with NP first. The wall is a dynamic and highly controlled structure [32,33], which is composed of carbohydrate polymers (cellulose, pectins, and hemicelluloses), lignin, and proteins in variable amounts [34]. As the first physical barrier, the cell wall determines whether NP can penetrate cells. To date, some studies have shown that NP can affect plant development by interacting with the wall without entering the plant cells [17,18,35]. This indicates that one of the reasons that could cause alterations in plant development is the physio-chemical modifications of the cell wall as a reaction to environmental changes [36,37,38,39,40,41]. This underlines the importance of cell walls research concerning the effects of NP on plants. A few reports have indicated that NP may influence the physical properties of the cell wall as they cause enlargement of wall pores [42,43]; however, changes in the cell wall chemistry under NP conditions have been poorly described [18].

One of the essential components that build the cell wall is a group of polysaccharides called pectins [39,44]. Pectins have been reported to be involved in many developmental processes from cell elongation to the reaction of plants to biotic and abiotic factors [33,37,45,46,47,48,49,50,51,52,53,54,55,56,57,58,59,60,61,62,63,64,65]. Despite such a large amount of research on the involvement of pectins in the response to stress conditions, there are still little data in relation to NP. It was shown that in Arabidopsis, the zero nanovalent iron induces the loosening of the cell wall [66] and that CuO NP caused physical damage and a biochemical disruption of the cell walls by loosening the tethers between the cellulose microfibrils [67]. Such findings indicate that the first reaction to NP might be remodeling the cell walls.

Other important components of the cell wall are the arabinogalactan proteins (AGPs) that belong to the subfamily of hydroxyproline-rich glycoproteins (HRGPs). They probably occur in every plant cell [68,69,70,71,72]. The presence of AGPs has been reported in both the cell walls and cytoplasmic compartments [73,74,75]. The AGPs are postulated to be involved in different developmental processes as well as in the reaction of plants to environmental factors [33,76,77,78,79,80,81,82]. Treating Arabidopsis with the zinc oxide (ZnO) NP showed the downregulation (among others) of the AGPs genes [83]. For Arabidopsis, it was also proven that AGP can be extracellular molecules for binding to exogenously applied cerium NP [84]. However, it is not known how treatment with NP affects the presence or distribution of different AGP epitopes. 

This study aimed to evaluate the effect of NP of various sizes and surface properties on cell wall chemistry. For this purpose, neutral gold NP with different diameters and surface charges were used. The research was conducted on the model crop plant *Hordeum vulgare* L. and the parts of the plant that were analyzed were the roots. The changes in the composition and distribution of selected pectic (LM5, LM6, LM8, JIM5, JIM7) and AGPs (JIM8, JIM13, JIM16, MAC207, LM2) epitopes were analyzed in various tissues of the root apex (RA), and the differentiation zone (DZ) of the roots using the immunohistochemical method. The results indicate that the cell wall chemistry undergoes various modifications in response to NP with different properties. This knowledge can provide valuable new data for understanding the mechanisms that underlie the interactions of NP with plants. Moreover, this is the first paper that analyses the changes in the chemistry of the cell wall under the influence of NP using such a broad range of antibodies.

## 2. Materials and Methods

### 2.1. Nanoparticles Characterisation

Gold nanoparticles (spheres) 5 nm and 20 nm in diameter were purchased from nanoComposix Europe, Prague, Czech Republic. The AuNP were coated with 1/polyethylene glycol (PEG), which neutralizes charge and improves NP stability and dispersion in a medium; 2/branched polyethyleneimine (BPEI) that contains the amino groups, which causes the formation of (+) AuNP and 3/citrate, which causes the formation of the (−) AuNP. The color of the nanoparticle solution is a shade of red.

### 2.2. Plant Material

The *Hordeum vulgare* L. cultivar Karat (variety ID: 1228; registration year 1981; mvd.iaea.org accessed on 15 July 2021) was used as the model crop plant to investigate the uptake of the AuNP. The seeds were derived from the collection of Iwona Szarejko’s team at the Institute of Biology, Biotechnology, and Environmental Protection, Faculty of Natural Sciences, University of Silesia in Katowice, Poland.

### 2.3. Culture and Treatment

The barley seeds were surface sterilized by submerging them in a 20% sodium hypochlorite solution for 20 min and rinse them three times with sterile distilled water for 5 min, and then left in water for 24 h (at room temperature, RT) for imbibition. Caryopses were put into Petri dishes filled with four layers of filter paper moistened with 3 mL of deionized water. Petri dishes were sealed by a plastic film to prevent evaporation and incubated at RT in dark (48 h). Next, the seeds with emerging radicles were cultivated hydroponically for 7 days in glass tubes that were closed with a sealing film strip (Parafilm^®^M, Bionovo, Legnica, Poland). The roots of the barley seedlings were grown in a 1/16 strength Hoagland nutrient solution (pH = 7) that had been enriched with 5 nm and 20 nm neutral AuNP, and 5 nm positively and 5 nm negatively-charged AuNP, all at a concentration 50 µg/mL (the volume of medium per plant was 20 mL). The color of the medium with AuNP did not change during the culture what indicated that the tested NP were stable. Aeration of the nutrient solution was not controlled. The plants were grown in a growth chamber under 16 h photoperiod conditions, 20 °C and 180 μE m^–2^ s^–1^ of light. The control plants were cultured under the same conditions but without the addition of AuNP. 

### 2.4. Sample Preparation

The control and the treated seminal roots (neutral 5 nm AuNP, neutral 20 nm AuNP, positively and negatively charged AuNP) of seven-day-old barley seedlings were harvested and subjected to a further procedure. The roots were fixed in a mixture of 4% paraformaldehyde (PFA), 1% glutaraldehyde (GA) in phosphate-buffered saline (PBS, pH = 7.2) overnight at 4 °C. Subsequently, the samples were washed three times with PBS for 15 min each, dehydrated in increasing ethanol concentrations (10, 30, 50, 70, 90, 100% EtOH in distilled water, 2 *×* 30 min each step) and gradually embedded in LR White resin (Polysciences, Warrington, PA, USA). Next, the samples were cut into 1.5 µm thick cross-sections for the root DZ and longitudinal sections for the root apex using an EM UC6 ultramicrotome (Leica Biosystems, Zalesie Gorne, Poland). The sections were placed on glass slides that had been coated with poly-L-lysine (Polysine^®^, Menzel Thermo Scientific, Jiangsu, China).

The sections were treated with 2% fetal calf serum and 2% bovine serum albumin in PBS (blocking buffer) for 30 min at RT to block any nonspecific binding sites. After blocking, the samples were incubated with the primary monoclonal antibodies (Plant Probes, Regensburg, Germany; see Table 1) and diluted 1:20 in a blocking buffer at 4 °C overnight. The sections were then washed with the blocking buffer (three times, 10 min each) and incubated with the secondary antibody conjugated with Alexa Fluor 488 goat anti-rat IgG (green fluorescence; Jackson Immuno Research Laboratories, West Grove, PA, USA) and diluted 1:100 in the blocking buffer for 1.5 h at RT. Next, the slides were washed with the blocking buffer (3 × 10 min) followed by staining with 0.01% fluorescent brightener 28 (calcofluor—binds to cellulose, applied in order to visualize cell walls, blue fluorescence; Sigma-Aldrich, Poznan, Poland) in PBS for 5 min. Subsequently, the samples were rinsed in PBS (3 *×* 10 min) and sterile distilled water (5 *×* 5 min). The dried slides were mounted with the anti-fading medium Fluoromount (Sigma-Aldrich). To confirm the specificity of a secondary antibody, negative controls were made in which the primary antibody step was omitted, and the blocking buffer was applied instead. Each negative control section exhibited no fluorescence signal (not shown). The observations and photographic documentation were carried out using an epifluorescence microscope (each section was photographed in two channels: for Alexa Fluor 488—excitation filter 450–490, barrier filter BA520; for CF—excitation filter 330–380, barrier filter BA420) Nikon Eclipse Ni-U microscope equipped with a Nikon Digital DS-Fi1-U3 camera with the corresponding software (Nikon, Tokyo, Japan). Each variant of the experiment and the staining were performed in three repetitions. The figures are composed of representative photographs that were obtained while documenting the distribution of the pectic and AGPs epitopes in the control and treated roots.

## 3. Results

### 3.1. Histological Characteristics of Barley Control Roots

**Figure 1:**
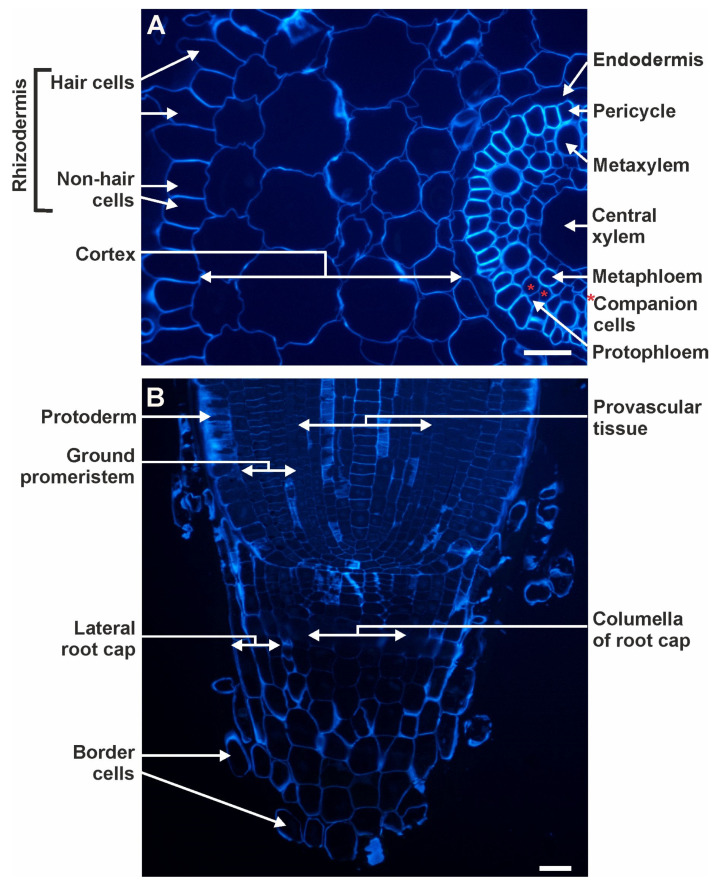
Representative images of the DZ (**A**) (cross-sections) and RA (**B**) (longitudinal sections) of the control barley roots cv. Karat. CF staining. * mark the companion cells. Scale bars = 25 μm.

In the cross-section of the DZ of the control roots, the following tissues could be distinguished: the rhizodermis, consisting of hair and non-hair cells; four layers of cortical cells; the endodermis; the pericycle; and the stele (Figure 1A). In a longitudinal section of the RA, the following tissues were present: the protoderm (in the text described as rhizodermis), the ground promeristem (in the text described as the cortex), the provascular tissue (in the text described as stele: endodermis, pericycle, phloem, xylem) and the root cap, which consisted of the columella as well as lateral and border cells (Figure 1B).

### 3.2. Immunohistochemical Analysis of the Changes in the Distribution of the Pectic and AGP Epitopes after AuNP Treatment

#### 3.2.1. Pectic Epitopes

The distribution of the pectic epitopes (LM5, LM6, LM8, JIM5, JIM7) in the DZ (Supplementary Table S1) and the RA (Supplementary Table S2) of the control and experimental roots were analyzed.

##### LM5 Epitope (Galactan Side Chain of Rhamnogalacturonan-I, RG-I; (1→4)-β-D-galactan)

**Figure 2:**
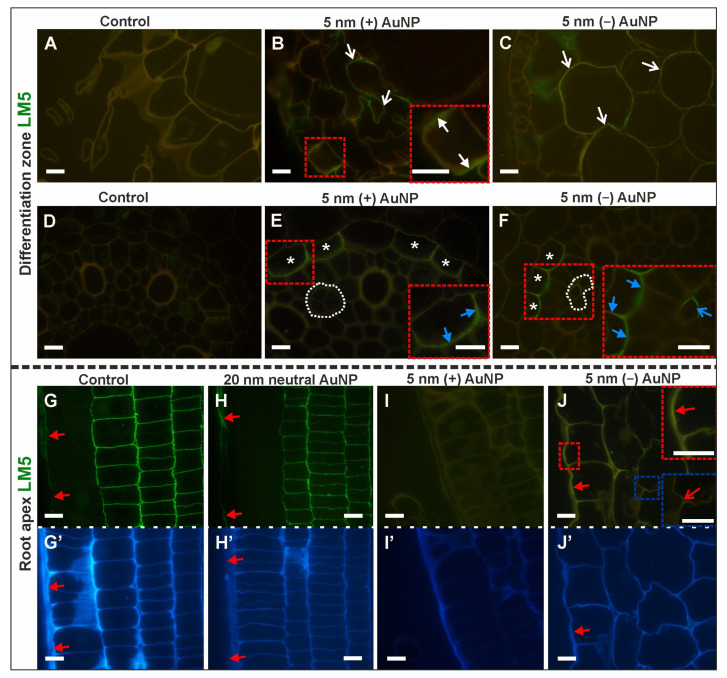
Differences in the distribution of the LM5 epitope in the DZ (rhizodermis and cortex, (**A**–**F**); cross-sections) and in the distal part of the RA (rhizodermis of the distal part of the root), (**G**–**J**); longitudinal sections between the control and treated roots. CF staining (**G’**–**J’**). White arrows indicate an LM5 signal in the anticlinal walls in the rhizodermis in the DF; open white arrows point to LM5 signal in the walls of the cortex in the DF; white asterisks mark the endodermal cells in the DF where the LM5 epitope was detected; blue arrows indicate the LM5 signal in the walls of the endodermis in the DF, white dotted line outlines the phloem cells in the DF, open blue arrow indicates the LM5 signal in the phloem cell in the DF; red arrows point to LM5 signal in the rhizodermis in the RA; open red arrow indicates an LM5 signal in the cortex in the RT. Red and blue dashed squares outline the locations of the enlargements in the insets. Scale bars = 10 μm.

The LM5 RG-I epitope distribution in the DZ was changed upon AuNP treatment (Figure 2A–F; Table 2; Supplementary Table S1).

It was not detected in any of the tissue in the control roots (Figure 2A,D) nor in those that had been treated with 5 nm and 20 nm neutral AuNP (Supplementary Table S1), however, the epitope was detected in the roots after being treated with charged AuNP (Figure 2B,C,E,F). In the roots that had been treated with (+) AuNP, LM5 occurred in the anticlinal walls of some rhizodermal cells and the individual cells of the cortex layer (Figure 2B). In these roots, the LM5 epitope was found only in the anticlinal and inner periclinal walls of the endodermal cells that were above the phloem field (Figure 2E). The same pattern of LM5 labeling in the endodermis was detected in the roots that had been treated with 5 nm (−) AuNP (Figure 2F). Moreover, in these roots, the LM5 antibody was present in the walls of some phloem cells (Figure 2F) and cortical cells but not in the rhizodermis (Figure 2C).

Differences in the distribution of the LM5 epitope were also found in the RA (Figure 2G–J,G’–J’—outline cells after calcofluor (CF) staining; Table 2; Supplementary Table S2). The LM5 epitope was present in the outer periclinal wall of the rhizodermis (Figure 2G,H,J). It also occurred abundantly in the cortex of the control roots (Figure 2G), the plants that had been treated with 5 nm and 20 nm neutral AuNP (Figure 2H), and in some walls of the cortical cells in roots that had been treated with (−) AuNP (Figure 2J, bottom inset). However, in the roots that had been treated with 5 nm (+) AuNP, there was no LM5 antibody signal in the rhizodermis or the cortex (Figure 2I). Additionally, in all of the analyzed roots, the LM5 epitope was detected within the walls of the lateral and border cells of the root cap.

To summarize, it can be stated that the pectic epitope, which is recognized by the LM5 antibody is not a constitutive component of the walls of cells of the DZ. Thus, the occurrence of LM5 in the cell walls from the roots that had been treated with NP might be a reaction to this factor. Conversely, in the RA, this epitope was present in the control roots but was absent from treated roots, which means that the reaction is to abort its synthesis in this zone. The obtained results also indicate the diverse presence of this component in the walls along the root zones in the control roots. Moreover, depending on the character of the cells, dividing versus differentiating, the occurrence of this epitope after NP treatment was different (Table 2).

##### LM6 Epitope (Arabinan Side Chains of RG-I; (1→5)-α-L-arabinan)

**Figure 3:**
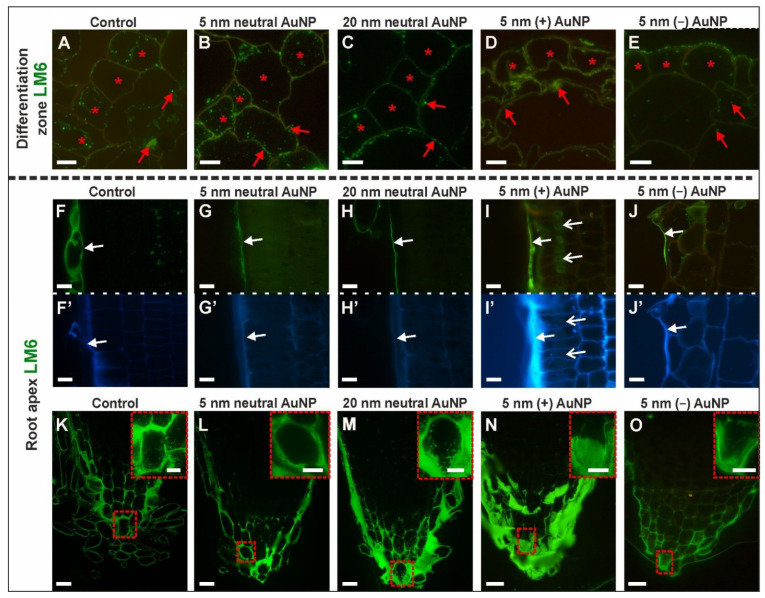
The distribution of the LM6 epitope in the DZ (rhizodermis and cortex, (**A**–**E**); cross-sections) and the RA (rhizodermis of distal part of the root, (**F**–**J**); root cap**,** (**K**–**O**); longitudinal sections) of the control and treated roots. CF staining (**F’**–**J’**). Red asterisks mark the rhizodermal cells in the DZ where the LM6 epitope was present; red arrows point to the LM6 signal in the cortex in the DZ; white arrows indicate the occurrence of the LM6 epitope in the outer periclinal cell wall of the rhizodermis in the RA; open white arrows indicate the presence of LM6 near the nucleus. Red dashed squares denote the locations of the enlargements in the insets. Scale bars A–J’, K–O insets = 10 μm; K–O = 25 μm.

The distribution pattern of LM6 was slightly changed by the AuNP treatment in the differentiation zone compared to the control roots (Figure 3A–E; Table 2; Supplementary Table S1). In all of the analyzed variants, the LM6 antibody was present in the intracellular compartments (in short, this term will be described as a signal in the cytoplasm) and the cell wall primarily in the rhizodermis as well as in some of the cortical cells (Figure 3A–E). The LM6 epitope was observed in the cell wall and cytoplasm of some of the endodermal cells, some of the pericycle cells, and most of the phloem cells regardless of the treatment (Supplementary Table S2).

The pectic epitope, which is recognized by the LM6 antibody, differed slightly in the RA among the analyzed variants (Figure 3F–O,F’–J’—outline cells after CF staining; Table 2; Supplementary Table S2). The signal was detected in the outer periclinal wall of the rhizodermis in all of the variants (Figure 3F–J). In the control roots, the LM6 epitope occurred in the cytoplasmic compartments in some of the cortical cells (Figure 3F), however, in the roots that had been treated with 5 nm and 20 nm neutral AuNP, this epitope was not observed in the cortex (Figure 3G,H). Moreover, in the roots that had been treated with (+) AuNP and (−) AuNP, it was detected in the cytoplasm of individual cells (Figure 3I,J). Furthermore, in the roots that had been treated with 5 nm (+) AuNP, the LM6 epitope was also present in the cytoplasm in the vicinity of the nucleus (Figure 3I). The LM6 epitope was present in the root cap cells in all of the analyzed roots (Figure 3K–O), especially in the walls and cytoplasm of the columella region, the lateral root cap cells, and border cells. The signal was also observed in the mucilage, which was very abundant in the roots that had been treated with 20 nm neutral AuNP (Figure 3M) and (+) AuNP (Figure 3N).

It can be concluded that surface-charged NP affects the wall composition and that this is manifested by a decreased presence of the LM6 epitope in the rhizodermis (compared to the control), while the neutral NP stimulated the synthesis of this epitope. In the roots that had been treated with the negatively-charged NP, there was a decrease in the number of exfoliated cells, which caused changes in the functioning of the root cap cells. Moreover, these NP caused a decrease in the synthesis of the LM6 epitope (Table 2).

##### LM8 Epitope (Xylogalacturonan (XGA), HG Domain)

The LM8 epitope was not detected in any of the analyzed tissues in the DZ regardless of the treatment (Supplementary Table S1). This epitope was found within the walls and cytoplasm of the lateral and border cells of the root cap only in the control roots and the plants that had been treated with (+) AuNP and (−) AuNP (Table 2).

##### JIM5 Epitope (Unmethylesterified/Low Methylesterified HG)

The JIM5 pectic epitope was only observed in the rhizodermis in the DZ in the roots that had been grown in the (+) AuNP solution (Table 2). In the other roots, this epitope was not detected in any of the analyzed tissues in the DZ or RA regardless of the treatment (Supplementary Table S1 and Table S2).

##### JIM7 Epitope (Methylesterified HG)

**Figure 4:**
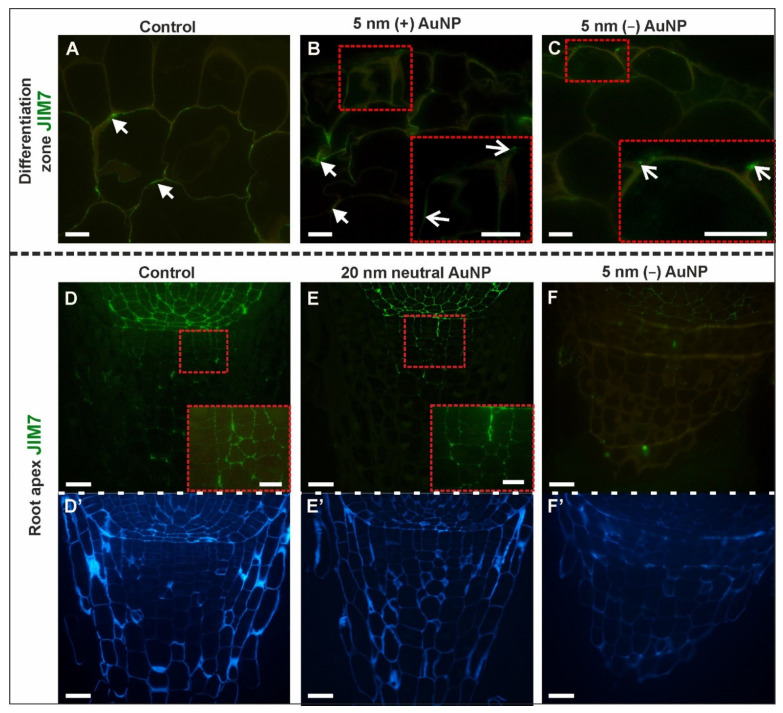
Presence of the JIM7 epitope in the DZ (rhizodermis and cortex, (**A**–**C**); cross-sections) and the RA (root cap, (**D**–**F**); longitudinal sections) in the control and treated roots. CF staining (**D’**–**F’**). White arrows indicate the occurrence of the JIM7 epitope in the cortex in the DZ; open white arrows show the LM6 signal in the rhizodermis in the DZ. Red dashed squares denote the locations of the enlargements in the insets. Scale bars A–C, B–E insets = 10 μm; D–F’ = 25 μm.

There were differences in the distribution pattern of the JIM7 epitope in the DZ, especially in the rhizodermis and cortex (Figure 4A–C; Table 2; Supplementary Table S1). A fluorescence signal was not observed in the rhizodermis of the control roots, (Figure 4A), roots treated with 5 nm and 20 nm neutral AuNP (not shown), however, a punctate signal was found in the walls of some of the rhizodermal cells in (+) AuNP (Figure 4B) and (−) AuNP (Figure 4C). In the cortical cells of the control roots and treated with 5 nm and 20 nm neutral AuNP and (+) AuNP, JIM7 was detected in some of the walls of individual cells (Figure 4A,B), but it was not observed in the (−) AuNP-treated roots (Figure 4C). In all of the analyzed variants, the JIM7 epitope was also present in the endodermal cells walls, pericycle, and phloem, but it was not observed in the xylem (Supplementary Table S1).

**Figure 5:**
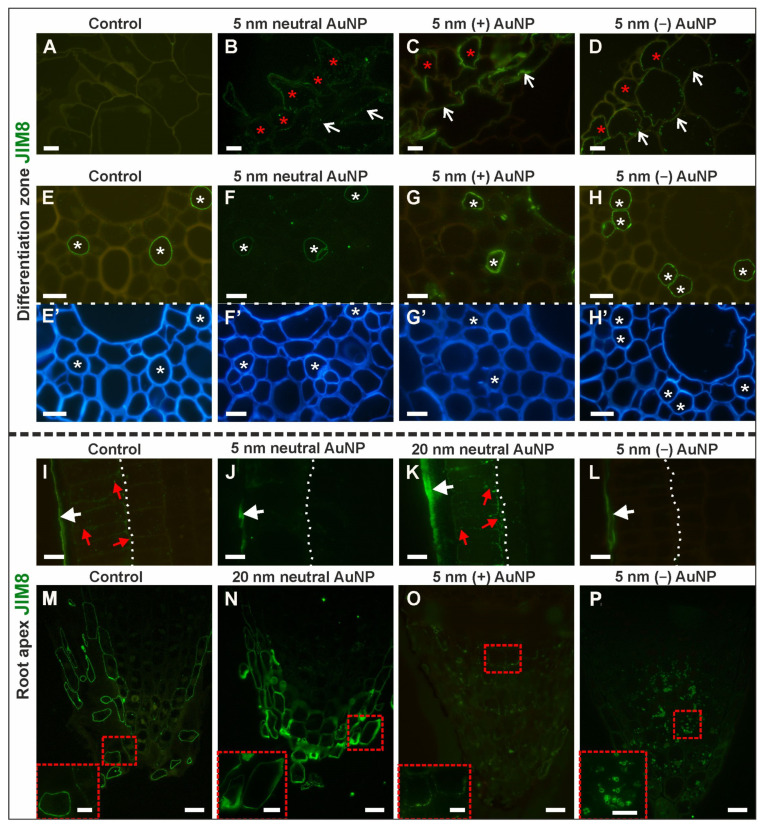
Changes in the distribution of the JIM8 epitope in the DZ (rhizodermis and cortex, (**A**–**D**); stele cells, (**E**–**H**); cross-sections) and the RA (rhizodermis of distal part of the root, (**I**–**L**); root cap, (**M**–**P**); longitudinal sections) between the control and treated roots. CF staining (**E’**–**H’**). Red asterisks mark the rhizodermal cells in the DZ where the JIM8 signal was detected; open white arrows indicate the occurrence of the JIM8 epitope in the cortex in the DF; white asterisks indicate the JIM8 signal in the metaphloem cells in the DZ; white arrows show the presence of the JIM8 in the outer cell wall of the rhizodermis in the RA; red arrows indicate the presence of JIM8 in the anticlinal and inner walls of the rhizodermal cells in the RA; white dotted line (**I**–**L**) outlines the boundary of the rhizodermis/cortex in the RA. Red dashed squares denote the locations of the enlargements in the insets. Scale bars A–L, M–P insets = 10 μm; M–P = 25 μm.

In the RA of the control and treated roots, the JIM7 epitope was detected in the walls of the cortex and stele cells, but it was not detected in the rhizodermal cells (Supplementary Table S2). The only difference was observed for the root cap (Figure 4D–F,D’–F’—outline cells after CF staining). In the roots that had been treated with (−) AuNP, this epitope was not detected (Figure 4F), however, it was found in the walls of the proximal part of the columella in the rest of the analyzed roots (Figure 4D, D inset, Figure 5E, E inset).

To summarize, this epitope was less represented within the cell walls of treated roots in the DZ compared to the control, which might indicate a decrease in its synthesis. In the RA, this epitope is a constitutive component of the walls of the stele cells. The impact of the NP was diverse, and was dependent on the NP charge, as the synthesis of the epitope decreased in the columella cells after (−) AuNP treatment (Table 2).

#### 3.2.2. AGP Epitopes

The distribution of the AGP epitopes (JIM8, JIM13, JIM16, MAC207, LM2) in the root tissues of the DZ (Supplementary Table S3) and the RA (Supplementary Table S4) were also analyzed.

##### JIM8 Epitope (AGP Glycan)

Immunolabelling with the JIM8 anti-AGP antibody revealed changes in the DZ of the analyzed roots (Figure 5A–H,E’–H’—outline cells after CF staining; Table 3; Supplementary Table S3).

In the rhizodermis, the JIM8 epitope was not detected in control (Figure 5A) or the roots that had been treated with 20 nm neutral AuNP. However, it was observed in the treated roots: in the cell walls and cytoplasm in most of the rhizodermal cells after treatment with 5 nm neutral AuNP (Figure 5B) and within the walls in some of the rhizodermal cells in the roots that had been treated with (+) AuNP (Figure 5C) and (−) AuNP (Figure 5D). Similarly, in the cortex, this epitope was detected only in the roots that had been treated with 5 nm AuNP, but the distribution pattern differed, and was dependent on the charge of the AuNP: neutral—the fluorescence signal was punctate and was detected in the wall and cytoplasm (Figure 5B), (+) AuNP—the signal was continuous and present only in the walls (Figure 5C) and (−) AuNP—a discontinuous fluorescence signal was found in the cytoplasm and walls (Figure 5D). Within the stele, the JIM8 epitope was only detected in the walls of the protophloem cells in both the control and treated roots (Figure 5E–H).

The distribution of the JIM8 epitope also differed among the tissues in the RA (Figure 5I–P; Table 3; Supplementary Table S4). A fluorescence signal was observed in the outer periclinal walls of the rhizodermis (Figure 5I–L). In the control and the roots that had been treated with 20 nm AuNP, this epitope was also observed in the anticlinal and inner periclinal walls as well as in the cytoplasm (Figure 5I–K). The JIM8 epitope was not detected or was less represented in the cortex and stele of the meristematic zone regardless of the treatment (Supplementary Table S4). In the root cap of the control and the 5 nm and 20 nm neutral AuNP-treated roots, the JIM8 antibody signal was detected in the walls of the lateral root cap cells and border cells (Figure 5M, N insets). However, in the roots that had been treated with (+) AuNP, this epitope was almost not present in the walls and cytoplasm of the columella root cap cells (Figure 5O, O inset). Interestingly, after the (−) AuNP treatment, the JIM8 epitope was present only in the cytoplasm in the columella cells (Figure 5P, P inset). There were spatial differences in the occurrence of the JIM8 epitope depending on the variant.

To summarize, the most pronounced reaction to NP concerned the root cap cells in which the 20 nm NP caused an intensive synthesis of the JIM8 epitope. After (−) AuNP treatment, the synthesis also increased, which was supported by the presence of an epitope in the cytoplasm. Conversely, the (+) AuNP limited the epitope synthesis (Table 3).

##### JIM13 Epitope (AGP Glycan, (β)GlcA1→3(α)GalA1→2Rha I)

**Figure 6:**
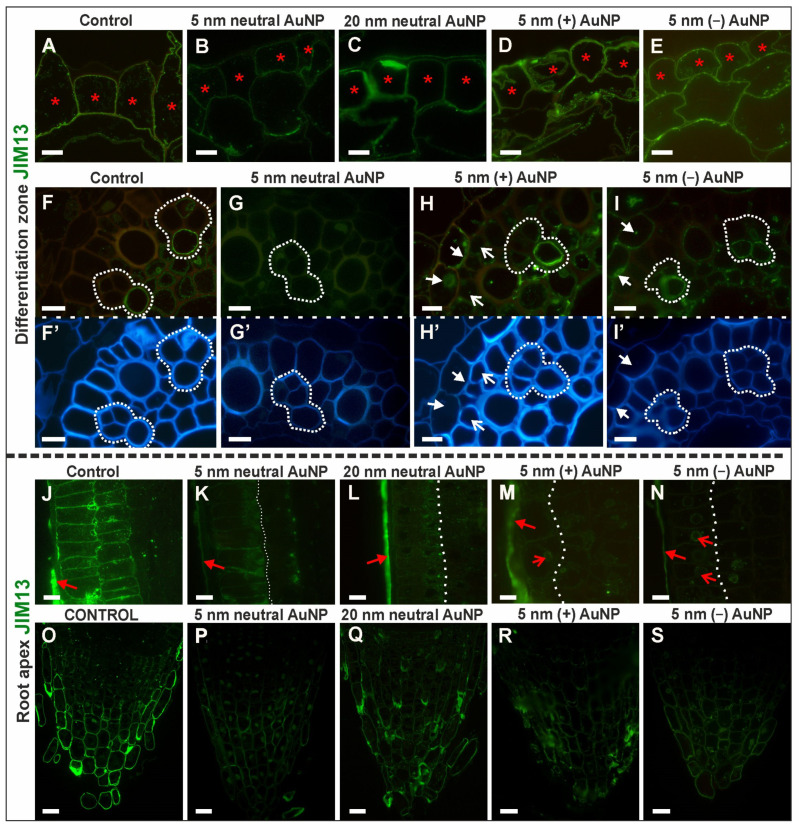
The distribution of the JIM13 epitope in the DZ (rhizodermis and cortex, (**A**–**E**); stele cells, (**F**–**I**); cross-sections) and RA (rhizodermis of distal part of the root, (**J**–**N**); root cap, (**O**–**S**); longitudinal sections) in the control and treated roots. CF staining (**F’**–**I’**). Red asterisks mark the rhizodermal cells in the DZ where the JIM13 signal was detected; white arrows show the endodermal cells where the JIM13 epitope was present in the DZ; open white arrows indicate the pericycle cells with the JIM13 epitope in the DZ; white dotted line (**F**–I**’**) outlines the protophloem, companion cells and metaphloem cells in the DZ; white dotted line (**K**–**N**) indicates the boundary of the rhizodermis/cortex in the RA; red arrows point to the presence of JIM13 in the outer cell wall of the rhizodermis in the RA; open red arrows indicate the occurrence of the JIM13 epitope near the nucleus in the rhizodermis in the RA. Scale bars A–N = 10 μm; O–S = 25 μm.

The distribution of JIM13 in the DZ tissues varied between the roots that had been treated with AuNP and the control (Figure 6A–I,F’–I’—outline cells after CF staining; Table 3; Supplementary Table S3). JIM13 was observed in the walls of the rhizodermis in all of the analyzed roots (Figure 6A–E). However, a punctate signal was also observed in the cytoplasm in the control roots and in the roots that had been treated with 5 nm neutral AuNP and (−) AuNP. In contrast, in the roots that had been treated with (+) AuNP and 20 nm neutral AuNP, the signal in the cytoplasm was rarely observed (Figure 6A–E). The JIM13 epitope was not detected in the endodermis in control (Figure 6F) or the roots that had been treated with 5 nm neutral AuNP (Figure 6G) and 20 nm neutral AuNP. However, it did occur in the wall and cytoplasm in some of the endodermal cells in the roots that had been treated with (+) AuNP and (−) AuNP (Figure 6H–I). The JIM13 epitope was not detected in phloem only in the roots that had been treated with 5 nm neutral AuNP (Figure 6G). In the other analyzed roots, the signal was observed in the walls in the phloem, especially in the metaphloem cells (Figure 6F,H,I). The JIM13 epitope was also present in pericycle cells but only in the roots that had been treated with (+) AuNP in which the fluorescence signal was localized in the cytoplasm (Figure 6H). This epitope was not detected in the metaxylem cells in any of the roots (Figure 6F–I).

##### JIM13 Epitope (AGP Glycan, (β)GlcA1→3(α)GalA1→2Rha I)

The distribution of JIM13 in the DZ tissues varied between the roots that had been treated with AuNP and the control (Figure 6A–I,F’–I’—outline cells after CF staining; Table 3; Supplementary Table S3). JIM13 was observed in the walls of the rhizodermis in all of the analyzed roots (Figure 6A–E). However, a punctate signal was also observed in the cytoplasm in the control roots and in the roots that had been treated with 5 nm neutral AuNP and (−) AuNP. In contrast, in the roots that had been treated with (+) AuNP and 20 nm neutral AuNP, the signal in the cytoplasm was rarely observed (Figure 6A–E). The JIM13 epitope was not detected in the endodermis in control (Figure 6F) or the roots that had been treated with 5 nm neutral AuNP (Figure 6G) and 20 nm neutral AuNP. However, it did occur in the wall and cytoplasm in some of the endodermal cells in the roots that had been treated with (+) AuNP and (−) AuNP (Figure 6H,I). The JIM13 epitope was not detected in phloem only in the roots that had been treated with 5 nm neutral AuNP (Figure 6G). In the other analyzed roots, the signal was observed in the walls in the phloem, especially in the metaphloem cells (Figure 6F,H,I). The JIM13 epitope was also present in pericycle cells but only in the roots that had been treated with (+) AuNP in which the fluorescence signal was localized in the cytoplasm (Figure 6H). This epitope was not detected in the metaxylem cells in any of the roots (Figure 6F–I).

Immunolabelling with the JIM13 antibody also resulted in differences in the RA among the analyzed roots (Figure 6J–S; Table 3; Supplementary Table S4). The JIM13 epitope was detected in the outer periclinal wall and cytoplasm of the rhizodermis in all of the examined roots (Figure 6J–N), especially in those that had been treated with 20 nm neutral AuNP (Figure 6L). However, only in the control roots, JIM13 was detected additionally in the anticlinal and inner periclinal rhizodermal cell walls (Figure 6J). Moreover, in the roots that had been treated with (+) AuNP and (−) AuNP, the fluorescence signal that was detected in the cytoplasm was localized near the nucleus (Figure 6M,N) and that localization was not observed for any of the other treatments. JIM13 was also present in the first layer of the cortex: in the walls and cytoplasm of the cortical cells in the control roots (Figure 6J) and in the cytoplasm of individual cells in the roots that had been treated with 5 nm neutral AuNP and (−) AuNP (Figure 6K,N). In the other roots, no fluorescence signal was detected in the cortex (Figure 6L,M). The JIM13 epitope was not detected in the stele in the analyzed roots except for the roots that had been treated with (−) AuNP in which the signal was hard to detect, indicating that its presence was significant (Supplementary Table S4). In the root cap, the JIM13 antibody was primarily present in the control roots (Figure 6O) and those that had been treated with 20 nm neutral AuNP (Figure 6Q) in which the fluorescence signal was localized in the cell walls of the lateral root cap, the border and some of the columella cells. In the roots that had been treated with 5 nm AuNP (neutral and charged), the presence of this epitope was reduced (indicated by the low intensity of the fluorescence; Figure 6P,R,S).

To summarize, the most pronounced response to NP was manifested in a reduction of JIM13 epitope synthesis in roots treated with neutral 5 nm NP in DZ. In RA, synthesis was reduced regardless of the NP type used (Table 3).

##### JIM16 Epitope (AGP Glycan)

**Figure 7:**
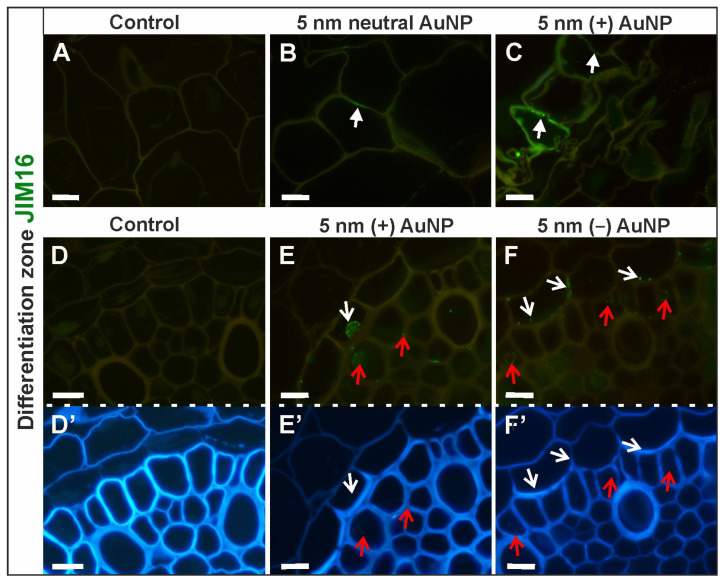
Differences in the distribution of the JIM16 epitope in the DZ (rhizodermis and cortex, (**A**–**C**); stele cells, (**D**–**F**); cross-sections) between the control and treated roots. CF staining (**D’**–**F’**). White arrows show the presence of the JIM16 epitope in the rhizodermis in the DZ; open white arrows indicate the JIM16 signal in the endodermis in the DZ; open red arrows point to the occurrence of JIM16 in the pericycle in the DZ. Scale bars = 10 μm.

In the DZ, JIM16 epitope distribution differed among tested roots (Figure 7A–F,D’–F’—outline cells after CF staining; Table 3; Supplementary Table S3). The epitope was not detected in the control roots (Figure 7A,D) and those treated with 20 nm neutral AuNP. In roots treated with 5 nm neutral AuNP, epitope was found within the walls in the single rhizodermal cells (Figure 7B) whereas in the roots treated with (+) AuNP the signal was present in the walls and cytoplasm in some of the rhizodermal cells (Figure 7C). Additionally, in the roots treated with (+) AuNP, a weak fluorescence signal was detected in the cytoplasm of individual endodermal (Figure 7E) and pericycle cells (Figure 7E). In the roots treated with (−) AuNP, the JIM16 presence was low in the cortex, endodermis (Figure 7F), and pericycle (Figure 7E).

JIM16 epitope did not occur in any tissue of the analyzed RA (Supplementary Table S4). In general, this epitope is not a constitutive wall component.

##### MAC207 Epitope (Arabinogalactan Protein, (β)GlcA1→3(α)GalA1→2Rha)

**Figure 8:**
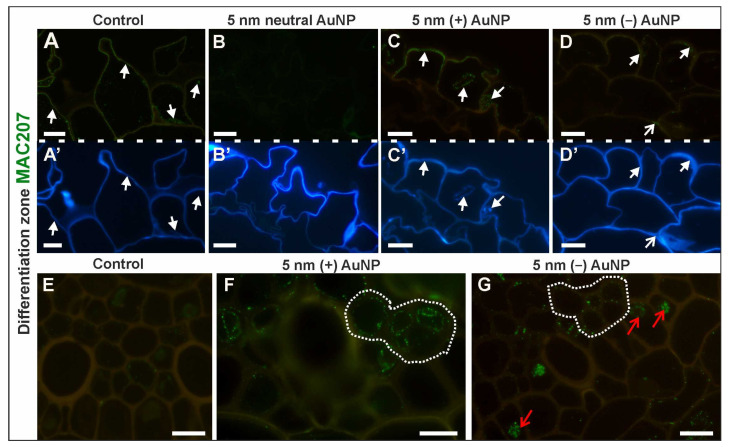
The distribution of the MAC207 epitope in the DZ (rhizodermis and cortex, (**A**–**D**); stele cells, (**E**–**G**); cross-sections) in the control and treated roots. CF staining (**A’**–**D’**). White arrows indicate the MAC207 signal in the rhizodermis in the DZ; open white arrow shows the MAC207 presence in the cortex in the DZ; white dotted line (**F**,**G**) outlines the protophloem, companion cells, and metaphloem in the DZ where the MAC207 signal occurred; open red arrows show the occurrence of the MAC207 epitope near the nucleus in the pericycle in the DZ. Scale bars = 10 μm.

In the DZ, the distribution of the MAC207 antibody differed among the analyzed roots (Figure 8A–G,A’–D’—outline cells after CF staining; Table 3; Supplementary Table S3). This epitope did not occur in any of the tissues of the roots that had been treated with 5 nm and 20 nm neutral AuNP (Figure 8B). In the control roots, the epitope was detected only in the cytoplasm and the walls of the rhizodermis as a punctate signal (Figure 8A). A similar pattern of MAC207 distribution was observed in the rhizodermis of the roots that had been treated with (+) AuNP (Figure 8C). In the roots that had been treated with (−) AuNP, the occurrence of the MAC207 epitope in the walls of the rhizodermis and the cortex was rarely detected (Figure 8D).

A weak signal of the MAC207 epitope was present in the cytoplasm of the phloem cells in the roots that had been treated with (+) and (−) AuNP (Figure 8F,G), however, the MAC207 epitope was detected in the cytoplasm of pericycle cells only in the roots that had been treated with (−) AuNP, and the signal was present near the nucleus (Figure 8E).

In the RA, the MAC207 epitope was only found in the roots that had been treated with (−) AuNP (Table 3; Supplementary Table S4). It was detected in the cytoplasm and outer periclinal wall of rhizodermis and the fluorescence signal was slightly observed in the cytoplasm of the columella cells in the root cap. 

Based on the results, it can be assumed that, at least in the root cap cells, this epitope is synthesized under the influence of NP.

##### LM2 Epitope (β -Linked GlcA)

**Figure 9:**
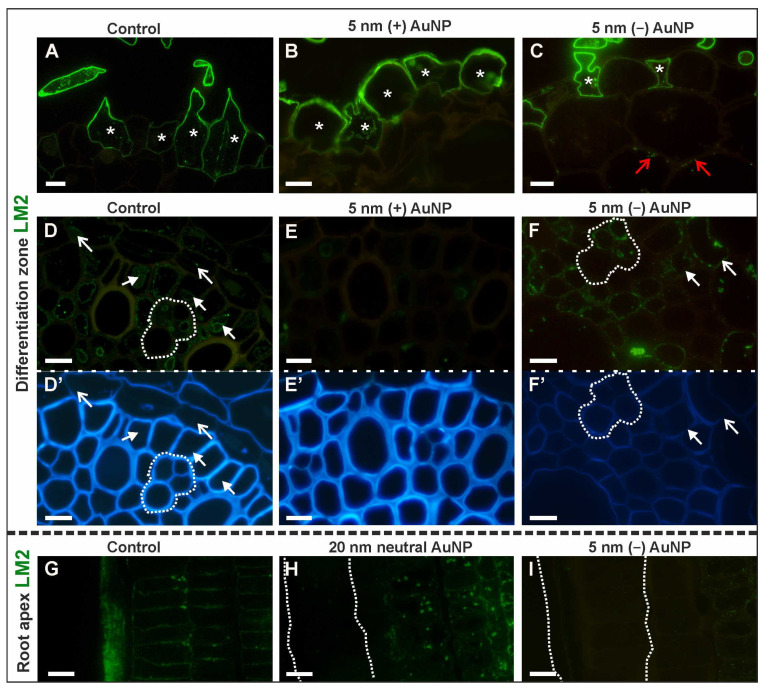
Presence of the LM2 epitope in the DZ (rhizodermis and cortex, (**A**–**C**); stele cells, (**D**–**F**); cross-sections) and RA (rhizodermis of distal part of the root, (**G**–**I**); longitudinal sections) in the control and treated roots. CF staining (**D’**–**F’**). White asterisks mark the rhizodermal cells in the DZ where the LM2 epitope was present; open red arrows show the LM2 epitope in the cortex cells in the DZ; open white arrows indicate the LM2 signal in the endodermis in the DZ; white arrows point to the presence of LM2 in the pericycle; white dotted line (D,D’,F,F’) outlines the protophloem, companion cells and metaphloem cells where the LM2 epitope was detected; white dotted line (H,I) denotes the rhizodermis in the RA. Scale bars = 10 μm.

There was also a difference in the occurrence of the LM2 epitope in the tissues in the DZ among tested roots (Figure 9A–F,D’–F’—outline cells after CF staining; Table 3; Supplementary Table S3). In the rhizodermis, this epitope was primarily present in the cytoplasm and the walls of hair cells in control (Figure 9A), and the roots that had been treated with neutral AuNP and (−) AuNP (Figure 9C). In the non-hair cells, the fluorescence signal was observed in individual cells (Figure 9A,C). In the roots that had been treated with (+) AuNP, this epitope was detected only in the walls and cytoplasm of the non-hair rhizodermal cells (Figure 9E; Figure 9E). The presence of the LM2 epitope in the cortex of (−) AuNP-treated roots was only slightly visible in the walls as a punctate signal (Figure 9C). In the control (Figure 9D) and roots that had been treated with neutral AuNP, the LM2 epitope was slightly present in the cytoplasm of the endodermal cells and was also found in the cytoplasmic compartments of some of the pericycle and phloem cells (Figure 9D). In the roots that had been treated with (−) AuNP, the LM2 antibody was observed in the cytoplasm and walls of the individual pericycle cells (Figure 9F) and some of the endodermal (Figure 9F) and phloem cells (Figure 9F).

To summarize, this epitope was primarily present in the hair cells in both the cell wall and cytoplasm. However, the non-hair cells that were undergoing anticlinal divisions were devoid of a fluorescence signal. Moreover, in the stele, (+) AuNP caused a decrease in the epitope synthesis (Table 3).

Differences in the occurrence of the LM2 epitope were also observed within the RA (Figure 9G–I; Table 3; Supplementary Table S4). The most pronounced differences were observed for the rhizodermis and cortex. In the control roots, the LM2 epitope was present in most of the rhizodermal cells (Figure 9G), while in the experimental roots, there was no fluorescence signal in this tissue (Figure 9H,I). Interestingly, LM2 was observed in the walls of the cortical cells in the control roots (Figure 9G), however, in the roots that had been treated with 20 nm AuNP, its localization was cytoplasmic in the cortical cells (Figure 9H). In the other examined roots, this epitope did not occur in the cortex. Additionally, in all of the analyzed variants, the LM2 epitope was detected within the walls of the lateral cells and border cells of the root cap and was also observed in the cytoplasm and walls of some of the cells in the stele. It can be assumed that in the roots that had been treated with 20 nm NP, this epitope was not synthesized in the rhizodermis and that the negatively-charged NP caused an epitope loss in most of the RA tissues.

## 4. Discussion

Plants are not able to react to harmful environmental factors by changing their locations as animals do. Instead, plants have developed a range of resistance mechanisms, that bear with abiotic stress [91,92]. In our research, we focused on the effect of NP on the chemical modifications in the cell wall as it is the first line of defense against external factors. The analyses were performed using a set of monoclonal antibodies against selected pectic and AGPs epitopes. The research showed that AuNP affected the cell wall chemical composition of barley root tissues. Moreover, changes in the presence and/or distribution of AGPs and pectic epitopes depended on the size and surface charge of the AuNP.

### 4.1. AuNP Affect the Presence and Distribution of Pectic Epitopes in Barley Roots

Pectins are structurally modulated in response to environmental conditions and abiotic stress [64,65,93,94,95,96,97]. Modifications in these wall polysaccharides are believed to affect the cell adhesion and the mechanical properties of plant tissues [98]. Our results demonstrate that AuNP treatment influenced the distribution of the LM5 and JIM7 epitopes in the DZ of barley roots as well as the distribution of the LM5, LM6, and LM8 epitopes in RA.

The LM5 and LM6 antibodies bind to carbohydrate residues within galactan and arabinan side chains of RG-I respectively [98]. Evidence suggests that walls rich in galactan are firm and stiff, whereas the abundance of arabinan provides wall elasticity [98,99,100,101]. Moreover, these two forms of RG-I side chains are believed to display a variable occurrence within the cell walls [102,103] and can be modified depending on different biotic and abiotic factors [65,104,105,106,107]. The presented research showed that the LM5 epitope was not detected in the tissues of the DZ in the control roots, but that it occurred in the walls of different tissues in the roots that were grown in (+) AuNP and (−) AuNP solutions. As it was demonstrated before, the changes in the occurrence of the LM5 epitope were correlated with a thickening of the outer rhizodermal walls [18]. Thus, the appearance of galactan in the cell walls of the tissues from the treated roots might cause wall stiffening and therefore, they could be a part of the reaction chains in the defensive response of plants to NP stress. In the RA, the difference in the distribution of the LM5 epitope was also observed for the roots grown in the (+) AuNP and (−) AuNP environments. In the control plants, LM5 was present in every tissue. However, this epitope was not detected within the walls of the rhizodermal or cortical cells after the (+) AuNP treatment and occurred only in the walls of some cells after the (−) AuNP treatment. The LM5 epitope has been shown to be involved in cell differentiation that takes place during plant development as it was detected in elongating cells, moreover, it may also be a marker of rapid cell growth [98,108,109,110,111,112]. These findings stand for the presence of galactan in the walls of the RA in the control roots. The presence of LM5 in the root cap, cortical cells, stele, and other root tissues was reported previously in carrots [111]. The absence of LM5 in the treated root apices might be correlated with the altered histological pattern of the root meristematic zone due to the (+) AuNP treatment, which was manifested, among others, by a decrease in the length of the meristematic zone and an increase in the radial cell dimensions of the cortical cells as was previously shown [18,65]. However, despite the growing number of studies, there is still no clear answer to how the presence or absence of the galactan side chains in the cell wall could affect its mechanical properties. After the treatment with 5 nm and 20 nm neutral AuNP, the LM6 epitope was not observed in the cortical cells in the RA. The occurrence of the arabinan side chain is associated with the rehydration of the cell walls [106] where arabinan itself might play the role of a pectic plasticizer to keep the cell wall flexible [99,101,106,113]. In the Arabidopsis root, it was found that the cell walls at the apical region appear to be arabinan-rich [98,114]. It was demonstrated that a reduction in the occurrence of arabinan epitope may be associated with an increase in cell wall stiffness [115]. Thus, the lack of the LM6 epitope in the walls in the cortical cells of the RA could cause structural changes that make the cell walls more rigid in AuNP conditions.

At this point, it is worth paying attention to the secretion of polysaccharides by the root cells, which has been indicated as being a reaction to stressful conditions. Root mucilage is mainly exuded from the outer layers of the root cap [116]. The increase in mucilage secretion by the roots that had been treated with NP that were observed in this study could be the result of plants reacting to stress. A similar observation was described for *Glycine max* roots growing under aluminum (Al) conditions [117]. The authors stated that the increased mucilage production surrounding root cap cells could be a unique response in developing a resistance to a toxic Al concentration. Similar results were described for rice [118]. Our results could indicate that the secretion of mucilage is a universal mechanism that is activated to protect the roots from harmful elements in the soil.

In comparison with dicotyledons, grasses are rather pectin and structural protein-poor plants [119,120]. In recent years, information on the chemical composition of the walls of grasses including crops has increased [97,121] but still, the roles of pectins and AGPs in monocot plants are being investigated. Therefore, the results presented here increase our knowledge about the presence of the pectic epitopes and changes in their spatial distribution under the influence of NP. Among others, our results indicate that the pectic epitopes that are recognized by the LM5, LM8, and JIM5 and the AGP epitopes that are recognized by JIM16 and MAC207 are not constitutive components of the walls of barley roots in either the DZ or the RA. When the presented information was compared with the literature [65], it can be concluded that the diversity in the constitutive components of grass walls is not only species-specific but is probably also specific to individual varieties, at least in barley.

HG is a major pectin that occurs in the primary walls of plants. HG is synthesized in the Golgi apparatus, but after its incorporation into the wall matrix, the polymer undergoes further local modifications. The extent of alternation (e.g., pattern or degree) affects the HG properties [122,123]. One of these modifications is the demethylesterification of HG that is catalyzed by pectin methylesterases (PMEs), which remove the methyl groups from the HG backbone. It has been shown on various organs (apical meristem, hypocotyl) that a reduction in wall stiffness was associated with increased pectin demethylesterification [49,124,125,126]. Our analysis of the distribution of the JIM7 epitope, that comprises partially methylesterified GalA residues [127], occurred in the rhizodermal walls in the DZ of the roots that had been treated with (+) AuNP and (−) AuNP compared to the control, where JIM7 was not observed. To date, it has been well documented that the degree of the methylesterification of HG plays a role in controlling cell growth and development [128]. As we demonstrated before, the barley rhizodermal cells have a changed phenotype under the influence of (+) AuNP [18]. Thus, our results could confirm a disturbing development and maturation of the rhizodermis as an effect of its interaction with AuNP.

The LM8 epitope, localized at the xylogalacturonan domain in HG, is reported to be associated with plant cell detachment [87]. To date, the LM8 epitope has been detected in the walls, intracellular compartments, and mucilage that covers the root cap cells in some angiosperm species [87,129,130]. Research on Arabidopsis roots revealed that LM8 specifically occurred in the outer surface of border root cap cells [131]. Our results are in accordance with this study as we found that the LM8 epitope was present in the lateral and border cells of the root cap in the control plants. Although the function of xylogalacturonan is not yet fully known, it is believed to play a role in plant defense. A high level of xylose substitution in the HG chain may prevent it from enzymatic digestion by pathogens, thus inhibiting or limiting the penetration of pathogens into the tissues [132]. However, in the roots that had been treated with neutral AuNP, the LM8 epitope was not detected. Rather, there was an increase in the secretion of mucilage (rich in LM6 epitope), which could compensate for the lack of XGA.

### 4.2. AuNP Affect the Presence and Distribution of the AGPs Epitopes in Barley Roots

The development of numerous signal transduction pathways, that involve the action of different classes of molecules, is another plant strategy against stress factors [76]. AGPs are believed to be among these molecules. AGPs are very complex glycoproteins that are involved in many developmental processes as well as the reaction of plants to biotic and abiotic factors [73,74,77,78,79,81,133,134,135,136,137,138,139]. Despite much research on AGPs, there is still no data related to the impact of NP on these macromolecules. In the present study, immunohistochemical analysis showed that AGPs may be involved in response to NP as their localization and/or presence were changed in the barley roots under the influence of AuNP. Changes were observed for the JIM8, JIM13, JIM16, MAC207, and LM2 epitopes in the DZ and the MAC207, JIM13, and LM2 epitopes in the RA.

We report an increase in the occurrence of the JIM8 epitope in the RA under different AuNP conditions and this observation coincides with previously presented works on Arabidopsis roots that had been treated with (+) AuNP [17]. Similar results were also observed in plants that had been subjected to salinity stress, e.g., in the leaves *of Medicago sativa* [140] or embryogenic suspension cultures of *Dactylis glomerata* L. [141]. Moreover, the changes in the abundance of the JIM8 epitope might be correlated with temperature stress [142,143,144]. Similarly, in the RA, we observed changes in the distribution of the JIM13, JIM16, and MAC207 epitopes mostly in the roots that had been treated with (−) AuNP and (+) AuNP. These epitopes occurred in various tissues of the treated roots in contrast to the control. An increase in the occurrence of the AGPs epitopes was also observed under temperature stress—the JIM13 and MAC207 epitopes in banana roots under chilling stress [143] and a more abundant occurrence of the LM2 epitope at a high temperature in Brachypodium leaves [142]. This may indicate that AGPs are involved in the response to unfavorable environmental conditions. It is worth mentioning that, in most cases, the appearance of the AGPs epitopes in the treated roots were found in the cytoplasmic compartments. This may be connected with the specific role of AGPs in the stress response to NP conditions and may indicate the activation of some signal transduction pathways. However, we also observed a decrease in the presence of some AGPs epitopes after AuNP treatment. In the roots that had been treated with (+) AuNP, we observed the absence of the above-mentioned LM2 epitope in most of the tissues from the DZ compared to the control roots. In the RA, the LM2 epitope did not occur in the rhizodermis in any of the treated roots compared to the control. AGPs are often observed as being associated with the endomembranes of plants cells. For example, the LM2 epitope was detected in the endoplasmic reticulum, Golgi apparatus, or Golgi-derived vesicles in maize root cells [145]. Thus, the absence of the AGPs epitopes in the AuNP-treated roots could indicate that the “natural state” was affected, which would indicate that this epitope could be a marker of plants during stress.

### 4.3. AuNP Presence Is Perceptible to Plant Cells

Although AuNP did not cross the barley rhizodermal wall [18] and did not penetrate the roots, the treatment resulted in a changed morphology and the wall composition of various cell types. Similar results were observed for Arabidopsis [17]. It is very intriguing how these particles, which are retained at the wall, affect processes inside a cell. A possible explanation could be NP interaction with cell wall-plasma membrane-cytoskeleton continuum, a hypothetical system that links the outside and the inside of plant cells (presented and reviewed in detail [146,147,148]), as it was suggested for another harmful factor—Al [149]. Al^3+^ can bind to negatively-charged pectins in the cell walls or exudates [150], which then immobilizes the ions. However, in maize roots, Al-induced structural modifications were observed in the cortical microtubules and the microfilament network [149]. These alternations depended on the examined root zone. In barley roots, Al changed the root histology and wall composition [65]. In the current study, a decrease in the thickness of the outer periclinal rhizodermal wall was observed in the roots that had been treated with (−) 5 nm AuNP as well as a change in the size and shape of the cells in the meristematic zone of the RA (unpublished, visible in Figure 2). Such alternations could possibly result from a modified cytoskeleton activity. Could pectins be a target for (+) AuNP binding? This is unlikely because un- or low-methylesterified HG (represented by the JIM5 epitope) was detected only in the DZ of the (+) AuNP treated roots, but not in the RA and DZ of the other variants; however, their impact on the roots was visible. (+) AuNP can also be bound by negatively charged molecules, for example, the glucuronoxylans from the hemicellulose group. In the case of (−) AuNP, we can point to the interaction with wall proteins with a positive charge such as extensins [34]. Neutral AuNP can interact with the wall components, but in a different kind of interaction as they have no charge. Although there are many unresolved questions and links to be solved in future studies, there is no doubt that plants can sense AuNP.

## 5. Conclusions

Changes in the chemical composition of the cell walls under the influence of abiotic stress are more and more frequently described in the literature and concern the influence of drought, salt, cold, heat, light (including UV radiation) stresses as well as heavy metals or air pollutants influence (for review see [41,104,106,151]). The obtained results revealed that also NP had a great impact on the changes in the chemical composition of the cell wall. It was demonstrated that depending on the physicochemical properties of NP, i.e., their size and surface charge, there were diverse changes in the distribution of the various pectic and AGPs epitopes in the tissues of the treated barley roots. Therefore, it can be concluded that one of the defensive and/or adaptive responses of plants to NP is a chemical alteration of the cell wall, which may, in turn, alter their physical properties. Thus, presented here results are a good addition to our knowledge about the mechanisms leading to the development of stress tolerance by plants. Moreover, it is possible that numerous modifications that are associated with the presence of the AGP epitopes may indicate the initiation of signaling pathways in response to NP.

## Figures and Tables

**Table 1:** List of the monoclonal antibodies used in the current study, the epitopes they recognized, and references. GalA—galacturonic acid, GlcA—glucuronic acid, Me-HG—methylesterified homogalacturonan (HG), Rha—rhamnose.

Antibody	Epitope
Pectins	
LM5	(1→4)-β-D-galactan [85]
LM6	(1→5)-α-L-arabinan [86]
LM8	xylogalacturonan (HG domain) [87]
JIM5	partially Me-HG/de-esterified HG [88]
JIM7	partially Me-HG [88]
Arabinogalactan Proteins	
JIM8	AGP glycan [75]
JIM13	AGP glycan, (β)GlcA1→3(α)GalA1→2Rha I [89]
JIM16	AGP glycan [89]
MAC207	arabinogalactan protein, (β)GlcA1→3(α)GalA1→2Rha [73]
LM2	Β-linked GlcA [90]

**Table 2:** Cellular response to AuNP treatment—changes in the presence of selected pectic epitopes in barley roots.

Pectins	Tissues	Neutral 5 nm	Neutral 20 nm	Positive 5 nm	Negative 5 nm
Differentiation Zone					
LM5	Phloem				ns
	Endodermis			ns	ns
	Cortex			ns	ns
	Rhizodermis			ns	
LM6	Phloem				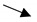
	Rhizodermis				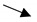
JIM5	Rhizodermis			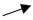	
JIM7	Phloem	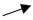	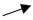	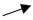	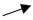
	Cortex				le
	Rhizodermis			ns	ns
Root Apex					
LM5	Cortex			le	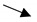
	Rhizodermis			le	
LM6	Cortex	le	le		
	Rhizodermis			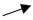	
	Root cap		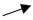	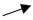	
LM8	Root cap	le	le		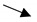
JIM7	Stele			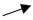	
	Root cap				le

ns—synthesis of new compounds in comparison to control; 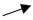 —increased presence in comparison to control; 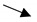
—decreased presence in comparison to control; empty cell—no distinct difference in comparison to control; le—lack of epitope; only those epitopes and tissues that exhibited changes in comparison to control are included in the table; details of results are presented in Supplementary Tables S1 and S2.

**Table 3:** Cellular response to AuNP treatment—changes in the presence of selected AGP epitopes in barley roots.

AGP	Tissues	Neutral 5 nm	Neutral 20 nm	Positive 5 nm	Negative 5 nm
Differentiation Zone					
JIM8	Phloem				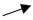
	Cortex	ns		ns	ns
	Rhizodermis	ns		ns	ns
JIM13	Phloem			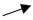	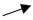
	Pericycle			ns	
	Endodermis			ns	ns
JIM16	Phloem				ns
	Pericycle				ns
	Endodermis			ns	ns
	Cortex				ns
	Rhizodermis	ns		ns	
MAC207	Phloem			ns	ns
	Pericycle				ns
	Cortex				ns
	Rhizodermis	le	le		
LM2	Phloem			le	
	Pericycle			le	
	Endodermis			le	
	Cortex				ns
	Hair cells			le	
	Non-hair cells			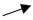	le
Root Apex					
JIM8	Rhizodermis	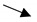		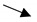	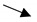
JIM13	Stele				ns
	Cortex		le	le	
	Rhizodermis	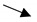	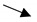	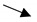	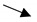
	Root cap	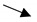		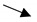	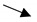
MAC207	Cortex				ns
	Rhizodermis				ns
	Root cap			ns	ns
LM2	Cortex	le		le	le
	Rhizodermis	le	le	le	le

ns—synthesis of new compounds in comparison to control; 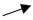
—increased presence in comparison to control; 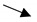
—decreased presence in comparison to control; empty cell—no distinct difference in comparison to the control; le—lack of epitope; only those epitopes and tissues that exhibited changes in comparison to control are included in the table; details of results are presented in Supplementary Tables S3 and S4.

## Data Availability

All the required data, which are related to the current study, are included in this manuscript.

## Supplementary Materials

The following are available online at https://www.mdpi.com/article/10.3390/cells10081965/s1, Table S1: Analysis of the distribution of the selected pectic epitopes in various tissues from the root DZ (based on cross-sections), Table S2: Analysis of the distribution of the selected pectic epitopes in various tissues in the RA (based on longitudinal sections), Table S3: Analysis of the distribution of the selected AGP epitopes in various tissues of the root DZ (based on cross-sections), Table S4: Analysis of the distribution of the selected AGP epitopes in various tissues in the RA (based on longitudinal sections).

## Abbreviations

AGP	arabinogalactan protein
AuNP	gold nanoparticles
(+) AuNP	positively-charged gold nanoparticles
(−) AuNP	negatively-charged gold nanoparticles
CF	calcofluor
DZ	differentiation zone
HG	homogalacturonan
NM	nanomaterials
NP	nanoparticles
RA	root apex
RG-I	rhamnogalacturonan I
XGA	xylogalacturonan
