# Supplementary material for: Gold Nanoparticles-Induced Modifications in Cell Wall Composition in Barley Roots
Source: Cells. 2021 Aug 2;10(8):1965. doi: 10.3390/cells10081965 (PMC8393560; doi:10.3390/cells10081965)
Supplement: Supplementary file 1 [file cells-10-01965-s001.zip › cells-1290516-supplementary.pdf]

cells MDPI

Supplementary Materials

Table S1. Analysis of the distribution of the selected pectic epitopes in various tissues from the root DZ (based on cross-sections). 3 – epitope present in 100% of the cells, 2 – epitope > 50% of the cells, 1 – epitope < 50% of the cells, 0 – epitope not detected; the values are derived from three samples of each type of tissue. Due to difficulty in identifying the separate fluorescence signals in the cell wall and plasma membrane at the light microscopy level, these two compartments are described together as a signal in the wall; signals detected inside the cells were classified as being localized in the intracellular compartments or the cytoplasm: C—cytoplasm, W—cell wall. Green indicates the most pronounced changes in the treated roots compared to the control.

Pectins		Control	Neutral 5 nm	Neutral 20 nm	Positive 5 nm	Negative 5 nm
LM5	Xylem	0	0	0	0	0
	Phloem	0	0	0	0	1 W
	Pericycle	0	0	0	0	0
	Endodermis	0	0	0	1 W	1 W
	Cortex	0	0	0	1 W	1 W
	Rhizodermis	0	0	0	1 W	0
LM6	Xylem	0	0	0	0	0
	Phloem	2 C, W	2 C, W	2 C, W	2 C, W	1 C, W
	Pericycle	1 C, W	1 C, W	1 C, W	1 C, W	1 C, W
	Endodermis	1 C, W	1 C, W	1 C, W	1 C, W	1 C, W
	Cortex	1 C, W	1 C, W	1 C, W	1 C, W	1 C, W
	Rhizodermis	2 C, W	2 C, W	2 C, W	2 C, W	1 C, W
LM8	Xylem	0	0	0	0	0
	Phloem	0	0	0	0	0
	Pericycle	0	0	0	0	0
	Endodermis	0	0	0	0	0
	Cortex	0	0	0	0	0
	Rhizodermis	0	0	0	0	0
JIM5	Xylem	0	0	0	0	0
	Phloem	0	0	0	0	0
	Pericycle	0	0	0	0	0
	Endodermis	0	0	0	0	0
	Cortex	0	0	0	0	0
	Rhizodermis	0	0	0	1 W	0
JIM7	Xylem	0	0	0	0	0
	Phloem	2 W	3 W	3 W	3 W	3 W
	Pericycle	1 W	1 W	1 W	1 W	1 W
	Endodermis	1 W	1 W	1 W	1 W	1 W
	Cortex	1 W	1 W	1 W	1 W	0
	Rhizodermis	0	0	0	1 W	1 W

Table S2. Analysis of the distribution of the selected pectic epitopes in various tissues in the RA (based on longitudinal sections). 3 – epitope present in 100% of the cells, 2 – epitope > 50% of the cells, 1 – epitope < 50% of the cells, 0 – epitope not detected; the values are derived from three samples of each type of tissue. Due to difficulty in identifying the separate fluorescence signals in the cell wall and plasma membrane at the light microscopy level, these two compartments are described together as a signal in the wall; signals detected inside the cells were classified as being localized in the intracellular compartments or in the cytoplasm: C—cytoplasm, W—cell wall. Green indicates the most pronounced changes in the treated roots compared to the control.

AGP		Control	Neutral 5 nm	Neutral 20 nm	Positive 5 nm	Negative 5 nm
JIM8	Xylem	0	0	0	0	0
	Phloem	1 W	1 W	1 W	1 W	2 W
	Pericycle	0	0	0	0	0
	Endodermis	0	0	0	0	0
	Cortex	0	1 C, W	0	1 W	1 C, W
	Rhizodermis	0	2 C, W	0	1 W	1 W
JIM13	Xylem	0	0	0	0	0
	Phloem	1 W	1 W	1 W	2 W	2 W
	Pericycle	0	0	0	1 C	0
	Endodermis	0	0	0	1 C, W	1 C, W
	Cortex	1 C, W	1 C, W	1 W	1 W	1 C, W
	Rhizodermis	3 C, W	3 C, W	3 W	3 W	3 C, W
JIM16	Xylem	0	0	0	0	0
	Phloem	0	0	0	0	1 C
	Pericycle	0	0	0	0	1 C
	Endodermis	0	0	0	1 C	1 C, W
	Cortex	0	0	0	0	1 C
	Rhizodermis	0	1 W	0	1 C, W	0
MAC207	Xylem	0	0	0	0	0
	Phloem	0	0	0	1 C	1 C
	Pericycle	0	0	0	0	1 C
	Endodermis	0	0	0	0	0
	Cortex	0	0	0	0	1 W
	Rhizodermis	1 C, W	0	0	1 C, W	1 C, W
LM2	Xylem	0	0	0	0	0
	Phloem	1 C	1 C	1 C	0	1 C, W
	Pericycle	1 C	1 C	1 C	0	1 C
	Endodermis	1 C	1 C	1 C	0	1 C, W
	Cortex	0	0	0	0	1 W
	Hair	3 W, C	3 W, C	3 W, C	0	1 C, W
	Non-hair	1 W, C	1 W, C	1 W, C	3 C, W	0

Table S3. Analysis of the distribution of the selected AGP epitopes in various tissues of the root DZ (based on cross-sections). 3 – epitope present in 100% of the cells, 2 – epitope > 50% of the cells, 1 – epitope < 50% of the cells, 0 – epitope not detected; the values are derived from three samples of each type of tissue. Due to difficulty in identifying the separate fluorescence signals in the cell wall and plasma membrane at the light microscopy level, these two compartments are described together as a signal in the wall; signals detected inside the cells were classified as being localized in the intracellular compartments or in the cytoplasm: C—cytoplasm, W—cell wall. Green indicates the most pronounced changes in the treated roots compared to the control.

Pectins		Control	Neutral 5 nm	Neutral 20 nm	Positive 5 nm	Negative 5 nm
LM5	Stele	1 W	1 W	1 W	1 W	1 W
	Cortex	3 W	3 W	3 W	0	1 W
	Rhizodermis	1 W	1 W	1 W	0	1 W
	Root cap	1 W	1 W	1 W	1 W	1 W
LM6	Stele	1 C	1 C	1 C	1 C	1 C
	Cortex	1 C	0	0	1 C	1 C
	Rhizodermis	1 W	1 W	1 W	2 C, W	1 C, W
	Root cap	1 C, W	1 W	2 C, W	2 C, W	1 C, W
LM8	Stele	0	0	0	0	0
	Cortex	0	0	0	0	0
	Rhizodermis	0	0	0	0	0
	Root cap	2 C, W	0	0	2 C, W	1 C
JIM5	Stele	0	0	0	0	0
	Cortex	0	0	0	0	0
	Rhizodermis	0	0	0	0	0
	Root cap	0	0	0	0	0
JIM7	Stele	1 W	2 W	1 W	2 W	1 C, W
	Cortex	2 W	2 W	2 W	2 W	2 W
	Rhizodermis	0	0	0	0	0
	Root cap	1 W	1 W	1 W	1 W	0

Table S4. Analysis of the distribution of the selected AGP epitopes in various tissues in the RA (based on longitudinal sections). 3 – epitope present in 100% of the cells, 2 – epitope > 50% of the cells, 1 – epitope < 50% of the cells, 0 – epitope not detected; the values are derived from three samples of each type of tissue. Due to difficulty in identifying the separate fluorescence signals in the cell wall and plasma membrane at the light microscopy level, these two compartments are described together as a signal in the wall; signals detected inside the cells were classified as being localized in the intracellular compartments or in the cytoplasm: C—cytoplasm, W—cell wall. Green indicates the most pronounced changes in the treated roots compared to the control.

AGP		Control	Neutral 5 nm	Neutral 20 nm	Positive 5 nm	Negative 5 nm
JIM8	Stele	0	0	0	0	0
	Cortex	0	0	0	0	0
	Rhizodermis	2 C, W	1 W	2 C, W	1 W	1 W
	Root cap	1 W	1 W	1 W	1 C, W	1 C
JIM13	Stele	0	0	0	0	1 C, W
	Cortex	1 C, W	1 C	0	0	1 C
	Rhizodermis	3 C, W	1 C, W	1 C, W	1 C, W	1 C, W
	Root cap	2 W	1 W	2 W	1 W	1 W
JIM16	Stele	0	0	0	0	0
	Cortex	0	0	0	0	0
	Rhizodermis	0	0	0	0	0
	Root cap	0	0	0	0	0
MAC207	Stele	0	0	0	0	0

	Cortex	0	0	0	0	1 C
	Rhizodermis	0	0	0	0	1 C, W
	Root cap	0	0	0	1 C	1 C
LM2	Stele	1 C, W	1 C, W	1 C, W	1 C, W	1 C, W
	Cortex	1 W	0	1 C	0	0
	Rhizodermis	2 W	0	0	0	0
	Root cap	1 C, W	1 C, W	1 C, W	1 C, W	1 C, W
